# The Impact of Metabolic Syndrome on Bone Mass in Men: Systematic Review and Meta-Analysis

**DOI:** 10.3390/biomedicines11071915

**Published:** 2023-07-06

**Authors:** Aleksandra Rył, Aleksandra Szylińska, Karolina Skonieczna-Żydecka, Tomasz Miazgowski, Iwona Rotter

**Affiliations:** 1 Department of Medical Rehabilitation and Clinical Physiotherapy, Pomeranian Medical University, Żołnierska 54, 71-210 Szczecin, Poland; aleksandra.ryl@pum.edu.pl (A.R.); iwona.rotter@pum.edu.pl (I.R.); 2 Department of Biochemical Sciences, Pomeranian Medical University, Władysława Broniewskiego 24, 71-460 Szczecin, Poland; 3 Department of Propaedeutics of Internal Diseases and Arterial Hypertension, Pomeranian Medical University, Powstańców Wielkopolskich 72, 70-111 Szczecin, Poland

**Keywords:** metabolic syndrome, osteoporosis, BMD, men, meta-analysis

## Abstract

Studies to date have yielded conflicting results on associations between components of metabolic syndrome (MetS) and bone mineral density (BMD), particularly in men. This current systematic review and meta-analysis addresses the existing gap in the literature and aims to evaluate bone mineral density (BMD) at the femoral neck (FN) and lumbar spine (LS) in men diagnosed with MetS. The two study authors independently searched PubMed, Cinahl, Embase, and Web of Science up to 8 February 2022 for studies in English. The inclusion criteria were (i) diagnosis of MetS according to the NCEP-ATP III 2001 criteria; (ii) adult male demographic; (iii) analyzable data on BMD in at least two sites using dual-energy X-ray absorptiometry (DXA), and (iv) original observational studies. Case reports and non-English articles were excluded. We analyzed the results of seven studies providing data on bone density in men with MetS. Results: Based on random effect weights, the mean BMD of the femoral neck and lumbar spine were 0.84 and 1.02, respectively. The mean lumbar spine T-score was −0.92. In meta-regression analysis, the variances in mean BMD in the lumbar spine and femoral neck could not be significantly explained by BMI (lumbar BMD: Q = 1.10, df = 1, *p* = 0.29; femoral neck BMD: Q = 0.91, df = 1, *p* = 0.34). Our meta-analysis suggests normal bone mass in adult males with MetS. Due to the high heterogeneity in the seven analyzed studies and the lack of control groups in these studies, further research is needed to fully elucidate the associations between MetS and its components and BMD in men.

## 1. Introduction

Metabolic syndrome (MetS) is a complex disorder defined as a cluster of interconnected cardiovascular risk factors. The global prevalence of MetS continues to rise, especially in urban populations of developed and developing countries [1]. Aside from environmental factors, lifestyle, and epigenetic influences, the MetS phenotype is also a significant deleterious determinant of cardiometabolic health and various MetS-related comorbidities such as polycystic ovary syndrome [2], benign prostatic hyperplasia [3], erectile dysfunction [4], proatherogenic lipid profiles [5], non-alcoholic fatty liver disease [6], hyperuricemia [7] and cancers [8]. In addition, constitutive MetS components, mainly attributable to obesity, have been suggested to be potential predictors of bone health. Although many studies have demonstrated a higher BMD and reduced risk of fractures in obese individuals in comparison to controls [9,10,11] or an inverted U-shaped relationship between BMI and BMD [12], it is generally believed that the reduced bone mass and increased fracture rates are associated much more with the level of adiposity and/or excessive visceral distribution of fat mass than with the surrogate measures of obesity, such as BMI or waist circumference (WC) [10,13,14]. Kim et al. found that high-density lipoprotein cholesterol (HDL-C) levels in serum are positively associated with trochanteric BMD; however, HDL-C levels were significantly lower in the control group than in the group with fractures [13]. Similar results were reported by Dimic et al. [15]. On the other hand, other studies demonstrated that HDL-C level was significantly higher in osteoporotic patients than in controls, and the risk of osteoporosis was significantly higher in women with a higher level of HDL-C [16,17]. Associations of BMD with serum triglycerides have been found to be positive [18], negative [15], and neutral [19].

Osteoporosis and MetS are two common multifactorial diseases characterized by a high incidence and prevalence in the adult population worldwide. Both are associated with high morbidity and mortality if not correctly diagnosed and accurately treated and also have a multifactorial pathogenesis characterized by a complex interplay between genetic and acquired factors, including unhealthy dietary habits such as high salt, high sugar, inadequate calcium intake, and low levels of physical activity. Osteoporosis, characterized by reduced bone mass, is a major risk factor for fractures, which often leads to increased morbidity and mortality. Osteoporosis affects women more often, but there is a rapid increase in the incidence of osteoporosis and fractures among men. In the US male population, increases in osteoporosis and osteopenia were observed in the 1990s (4%) and in 2000 (38%) [20]. The high morbidity and mortality associated with fractures and the increasing variety of pharmacological agents with clear benefits in reducing the risk of these fractures have made it increasingly important to identify men at increased risk of reduced bone mass. A study conducted among 234 asymptomatic men aged over 60 years was evaluated using radiographs and showed morphometric vertebral fractures in 32% of cases. Early identification of men with osteoporosis and a prompt introduction of pharmacological treatment will contribute to the prevention of fractures and thus reduce mortality [21]. Peak BMD in men is 8–10% higher than in women. In general, men have an increased bone size with more trabeculae than women, which provides a mechanical advantage, and this is why men lose bone mass later. The increase in bone diameter is associated with the action of androgens. Mechanical and hormonal advantage occurs in men; however, the aging of the body occurs in both genders, a vitamin D deficiency is related to diet and less exposure to the sun, and a decrease in muscle mass contributes to an increased risk of falls and thus to a greater risk of fractures. In recent decades, the incidence of osteoporosis and fractures has increased more strongly in men than in women. Between the early 1990s and mid-2000s, the incidence of osteoporosis and osteopenia in men over 50 years of age doubled [20].

More consistent results have been yielded by studies evaluating the associations of BMD with the constituent components of MetS. The majority of reports did not find an association between fasting blood sugar and BMD [15,22,23], but a Mendelian randomization study demonstrated that fasting glucose might be a causal risk factor for a smaller bone area at the hip, which in turn may artificially increase hip BMD [22]. Nonetheless, it is believed that in type 2 diabetes, despite a commonly normal BMD, the risk of osteoporosis and bone fractures is greater than in non-diabetics [24]. Several other reports have demonstrated inverse associations of BMD with the presence of hypertension, as well as the level of systolic and diastolic blood pressure in individuals both with and without hypertension [15,25].

In studies evaluating BMD in patients with MetS, the associations have been divergent—positive [26], neutral [27], inverse [28], positive in men and negative in women [29], and positive in women, while neutral in men [30].

This systematic review and meta-analysis address the existing gap in the literature and aims to evaluate BMD at the femoral neck (FN) and lumbar spine (LS) in adult men with MetS.

## 2. Materials and Methods

### 2.1. Search Strategy and Inclusion Criteria

At least two independent authors (AR and AS) searched PubMed, Cinahl, Embase, and Web of Science up to 8 February 2022 for observational studies in English evaluating bone health in adult men diagnosed with MetS.

The search strings are listed in Table 1.

The search was supplemented by a manual review of reference lists from eligible publications and relevant reviews.

The inclusion criteria were as follows:

1. Diagnosis of MetS according to the NCEP-ATP III 2001 criteria (age- and ethnicity-specific diagnostic criteria [31,32]);
2. Adult age and male sex;
3. Meta-analyzable data on BMD evaluated in at least 2 sites using dual-energy X-ray absorptiometry (DXA) in original studies.

The exclusion criteria were:

1. Review articles, commentaries, editorials, and letters to editors;
2. Case reports, case series (<10 patients);
3. Non-English articles.

### 2.2. Data Abstraction

Data on the study design, risk of bias [33], patient characteristics, and comorbidities from each study were independently extracted in accordance with the Preferred Reporting Items for Systematic Reviews and Meta-Analyses (PRISMA) standard by two independent investigators (AR and AS). Whenever data were missing for the review, the authors were contacted for additional information. Inconsistencies were resolved by consensus with a senior author (I). In case-control studies, data from only one study arm (MET patients) was abstracted.

### 2.3. Outcomes

Primary outcome measures were LS and FN BMD and LS and FN T-score. All of the outcomes were not, however, reported in all studies we included. Thus, the overall number of studies does not match number of studies depicted in particular forest plots.

### 2.4. Data Synthesis and Statistical Analysis

We conducted a random-effects [34] meta-analysis of outcomes for which ≥ 2 studies contributed data, using Comprehensive Meta-Analysis V3 (http://www.meta-analysis.com, accessed on 1 July 2022). We explored study heterogeneity using a chi-square test of homogeneity, with *p* < 0.05 indicating significant heterogeneity. All analyses were two-tailed with alpha = 0.05.

For continuous outcomes, we analyzed the pooled means in either endpoint scores (preferred) or changes from baseline to endpoint using the observed cases. Categorical outcomes were analyzed by calculating the pooled event rate. We conducted subgroup and exploratory maximum likelihood random-effects meta-regression analyses of the co-primary outcomes with BMI as the only variable. Finally, we inspected funnel plots and used Egger regression tests [35] and Duval and Tweedie’s trim and fill method [36] to quantify whether publication bias could have influenced the results.

Our analyses were characterized by high heterogeneity of the analyzed studies and the lack of control groups in the studies.

## 3. Results

### 3.1. Search Results

**Figure 1:**
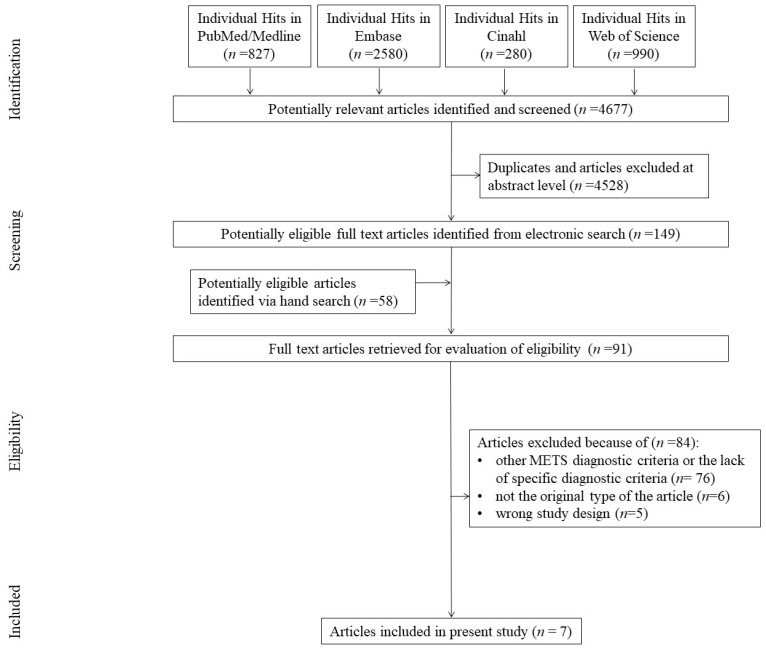
Flow chart.

The initial publication database search yielded 4677 studies, of which 4518 were excluded following evaluation on the title/abstract level or as duplicates. A total of 159 additional articles were identified via a manual search, of which 68 were duplicates of the search results, and 84 were further excluded due to not meeting the inclusion criteria. Finally, seven studies were analyzed. Primary reasons for exclusion were the use of other MetS diagnostic criteria or the lack of specific diagnostic criteria (n = 76), non-original type of study (n = 6), and inaccurate study design (n = 5) (Figure 1).

### 3.2. Study, Patient, and Regimen Characteristics

Altogether, seven observational studies involving 3533 male patients were included. The mean age of the study participants was 58.31 ± 9.89 years. Two studies were conducted in Saudi Arabia [37,38], and one study each from Iran [39], Brazil [40], South Korea [41], Belgium [42], and Taiwan [29]. All of these patients had been diagnosed with MetS. Data on MET-related anthropometric and biochemical parameters are shown in Table 2. In three studies [38,39,42], there was also information on bone health parameters in control groups (without a MetS diagnosis); however, only two [39,42] provided data on FN and LS BMD, as well as total hip BMD. In the study by Laurent, M. R. et al. [42], parameters were provided in a non-meta-analyzable form (median (Me) and interquartile ranges (IQRs)); thus, we only present data as single-group meta-analysis, which further limits the possibility of finding a direct link between MetS and BMD.

### 3.3. Risk of Bias

The mean score in the STROBE assessment tool was 25.57 ± 2.77 points (median = 26, Min = 20, and Max = 29). Overall, there was no study with the highest possible score. Details are presented in Supplementary Table S1.

### 3.4. Mean BMD in Patients with MetS

**Figure 2:**
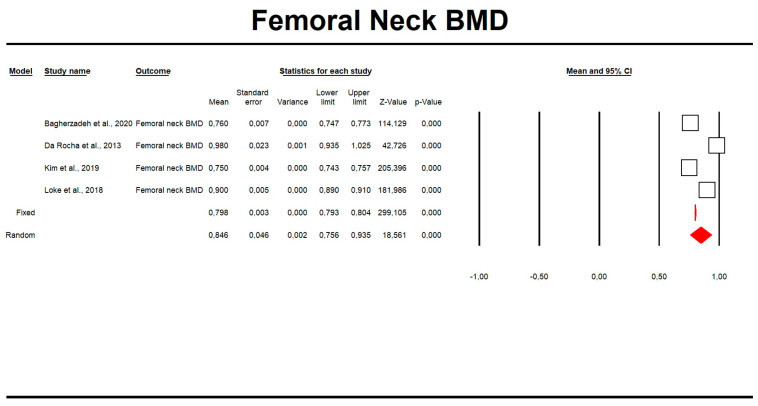
Forest plot of mean femoral neck BMD.

**Figure 3:**
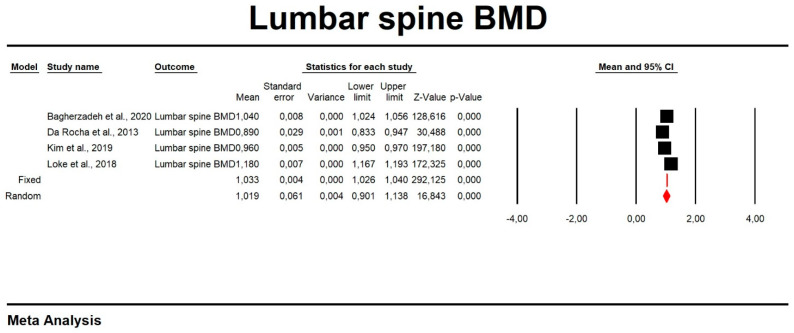
Forest plot of mean lumbar spine BMD.

**Figure 4:**
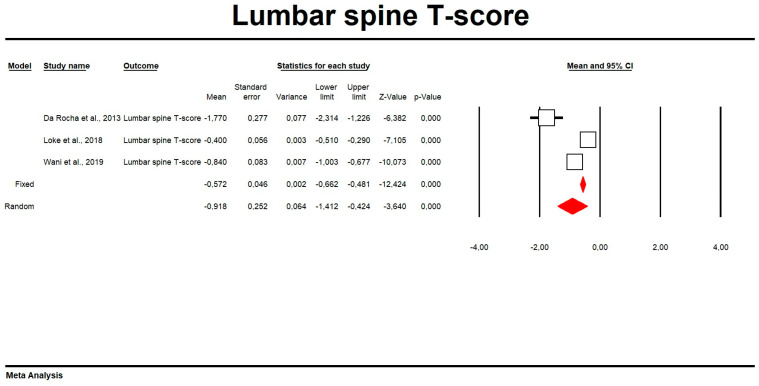
Forest plot of mean lumbar spine T-score.

Using random-effects weights, the overall means for FN and LS BMD were 0.84 and 1.02, respectively (Table 3, Figure 2 and Figure 3). The mean LS T-score was −0.92 (Table 3, Figure 4). No publication bias was detected (Egger’s test *p* > 0.05; funnel plots are presented in Supplementary Figure S1). In the meta-regression analysis, variances in mean FN and LS BMD in men with MetS were not significantly explained by body mass index (LS BMD: Q = 1.10, df = 1, *p* = 0.29; FN BMD: Q = 0.91, df = 1, *p* = 0.34). Scatterplots for the tested parameters were also made (Supplementary Figure S2).

## 4. Discussion

In our meta-analysis of seven qualified studies, the mean FN BMD was 0.84, the mean LS BMD was 1.019, and the mean LS T-score was −0.918, which strongly suggests normal bone mass in patients with MetS. Similarly, the study by Eckstein et al. [30] found no association between MetS and BMD in men (although it did in women). The study by Zhou et al. found a negative association of MetS with BMD in men [43]. In contrast, the study by Loke et al. demonstrated a positive association between MetS and BMD in men and a negative one in women. In addition, it was shown that an increase in the number of variables used to diagnose MetS significantly increased the positive association with BMD, even after adjusting for age [29]. In the study by Bagherzadeh et al., BMD measured at three sites (LS, FN, and total hip) was normal and positively associated with MetS. This association remained significant even after adjusting for the body mass index [39].

Similarly, other studies showed positive associations between weight and BMD [44,45]. Walsh et al. suggested the association might be related to an in vivo mechanism that includes increased leptin secretion by adipocytes and higher aromatase activity. In addition, they showed that visceral adipose tissue produces cytokines that increase bone resorption, which negatively influences bone strength [45]. These studies may suggest that obese people are usually at a lower risk of spine and proximal femur fractures but at a normal to slightly higher risk of fractures of the ankle and proximal humerus. In the study by Evans, it was concluded that obese people are protected from bone loss, an effect that decreases with age [44]. Positive associations between central obesity and BMD in men were also found [29,46]. However, the study by Jankowska et al. suggests the opposite, reporting a negative correlation between bone mass and visceral obesity [47]. In contrast, in our meta-regression analysis, LS and FN BMD were not related to body mass index.

Wang et al. [48] examined the relationship between metabolic obesity and forearm bone mineral density (BMD) in a general Chinese population divided into four groups: a metabolically healthy group with normal body weight, a metabolically healthy group with obesity, a metabolically unhealthy group with normal weight, and a metabolically unhealthy obese group. Men in the metabolically healthy obese group and women in the metabolically healthy normal-weight group were more likely to have lower forearm BMD at a younger middle age. They suggested the presence of metabolic obesity to be a better predictor of bone health than BMI alone and demonstrated that waist circumference, LDL-C concentration, and insulin resistance might be negative determinants of bone health.

The relationship between MetS and BMD was also examined in various disease states, but the results of these studies have been inconsistent. Wung et al. [49] found that MetS and all its individual components except high blood pressure were significantly associated with high lumbar spine and total hip T-scores, while BMI was positively associated with BMD in patients with MetS but not in those without MetS. In a recent study on BMD and bone structure in overweight men with diabetes (T2D) or MetS, Starup-Linde et al. [50] found that BMD at the hip was significantly lower in type 1 diabetes compared to MetS; however, the mean BMI value in type 1 diabetes was also significantly lower in comparison to type 2 diabetes and MetS. They found no differences in BMD measured at other sites (femoral neck, lumbar spine, and forearm), nor markers of bone turnover, nor in the majority of bone microarchitecture parameters between type 1 and type 2 diabetes and MetS. Du et al. [51] in Mendelian randomization analysis using large genome-wide association study (GWAS) summary statistics to assess the causal relationship between central obesity traits and BMD. They found that BMI-adjusted hip circumference was negatively correlated with BMD, while BMI-adjusted waist-to-hip ratio was positive, suggesting that waist circumference—a major component of MetS—could be a useful measurement also in the assessment of bone health. On the other hand, a study performed on a group of adolescents (10 to 16 years) with excess weight demonstrated reduced BMD in MetS [52].

Several studies evaluated the risk of osteoporosis in MetS. In 880 Caucasian men with MetS [53], there was no association of MetS with the prevalence of osteoporosis. In contrast, Rhee et al. [54] in a Korean population found that MetS was associated with a low occurrence of osteoporosis. However, in this study, MetS was positively associated with the occurrence of osteoporosis in both obese men and postmenopausal obese women. On the other hand, the recent meta-analysis by Babagoli et al. [55] demonstrated that metabolic syndrome had a protective impact on bone fracture rates in males but no net effect on fractures in females.

In the present study, further analyses of biochemical indices of MetS were not possible as they were either not reported or presented in mixed units. The study by Kim et al. in men showed negative correlations between femoral neck BMD and serum insulin and insulin resistance index (HOMA-IR), but no statistically significant levels were reported [41]. Similarly, a statistically insignificant correlation was found between BMD and HOMA-IR and serum insulin levels in the study by Aðbaht [56]. In contrast, the study by Basurto-Acevedo et al. showed a negative correlation of osteocalcin (a marker of bone formation) with insulin and HOMA-IR [57]. At a physiological level, insulin is known as an anabolic agent for bone formation [58]. However, insulin resistance observed in type 2 diabetes may weaken the physiological effects of insulin on bones [59], leading to increased porosity of cortical bone tissue and other changes in bone microarchitecture. In normal-weight individuals with MetS, even an elevated insulin concentration was associated with increased bone mineralization [54], suggesting different effects of this hormone on bone tissue between diabetic and non-diabetic individuals.

Other studies assessed the associations of BMD with lipid profiles. Some of them demonstrated that HDL-cholesterol was negatively correlated with BMD in men [18,29,56,60]. Others, in turn, showed no significant associations of BMD with LDL-cholesterol and triglycerides (TG) [18,29,56], while Adami et al. reported a positive correlation with LDL-cholesterol, total cholesterol, and TG [60].

There may be a relation between osteoporosis and cardiovascular disease as they share many common risk factors, such as increasing age, genetic factors or smoking. Evidence shows that people with cardiovascular disease have a 1.69 times higher risk of developing osteoporosis than people without cardiovascular disease [61]. Patients with osteoporosis were 1.2 to 1.4 times more likely to experience a cardiovascular event than non-osteoporotic patients [62]. Regardless of consistent or conflicting results, the positive association between lipid levels and osteoporosis can be explained by multiple biological mechanisms. Higher lipid levels were associated with increased oxidized lipids and higher oxidative stress. Higher oxidative stress can inhibit the differentiation of osteoblasts and promote the differentiation of adipocytes. In addition, the nuclear hormone receptor peroxisome proliferator-activated receptor gamma (PPARγ) may play a role in the relationship between BMD and lipid biomarkers. PPARγ can be activated by lipid metabolites. Osteogenesis is inhibited when PPAR-γ levels are elevated; this results in increased bone loss. The next mechanism is based on the fact that higher serum TG levels can be positively associated with higher marrow fat [38], which results in lower trabecular BMD [63]. The relationship between type 2 diabetes and the risk of osteoporosis is described in the literature. T2D and low-trauma fractures become more common with age. T2D is associated with higher BMD and greater body weight. While both have been historically believed to prevent fractures, paradoxically, the risk of fractures increases. This has been observed in T2D, although the risk is generally lower than in type 1 diabetes [64]. The Health, Aging, and Body Composition Study found that T2D was associated with higher hip, total body, and volumetric spine BMD independently of body composition and fasting insulin levels. The same cohort found an increased risk of fracture in diabetic patients, even after adjusting for age, calcaneal BMD, BMI, and other covariates. Risk factors for T2D fractures include older age, lower BMI, lower BMD, etc. Similar to type 1 diabetes, both diabetes duration and diabetes complications are associated with increased risk. The association between diabetes and fractures may also vary by population [64]. Looker et al. [65] find that associations between fracture risk and diabetes vary significantly by race/ethnicity. There are not many scientific studies on the direct relationship of hypertension itself with the risk of developing and developing osteoporosis. These studies are rather combined with the study of the relationship between osteoporosis and cardiovascular disease. A study by Hao Chai et al. [66] showed that Chinese postmenopausal women with osteoporosis had a higher prevalence of hypertension. Hypertension was significantly associated with osteoporosis.

Our study has some limitations. Due to the presence of various diagnostic criteria of MetS, only limited data were selected for this meta-regression. In addition, we narrowed diagnostic criteria for MetS to NCEP-ATP III 2001 guidelines, which in turn decreased the number of suitable reports and made it impossible to analyze the effect of certain MetS components. Due to the small amount of data reported in selected papers, it was not possible to conduct meta-regression analyses with all components of MetS. Additionally, since only one study provided adequate data for control groups (i.e., expressed as means and standard deviations), we chose to present only a single-arm meta-analysis, which limited us in our search for further associations between MetS and BMD. We tried to overcome this limitation by comparing these parameters with normal values. This meta-analysis was not registered in the PROSPERO database.

## 5. Conclusions

In conclusion, our meta-analysis suggests normal bone mass in males with MetS. However, due to the high heterogeneity of the analyzed studies and the lack of reports with control groups, further research is needed to fully elucidate the potential associations between MetS and its components and BMD in men.

There are many studies showing the protective effect of increased body weight on bone mass. However, in the conducted analysis, no such relationship was found in men. It is worth emphasizing that these analyses most often concern perimenopausal and postmenopausal women. It may seem that there may be different mechanisms influencing this relationship in women and men. Research on this topic should be developed, and new factors influencing bone mass in men should be sought. It is also worth taking into account such factors as the influence of gender, eating habits and lifestyle, and hormonal balance.

## Figures and Tables

**Table 1:** Search strings utilized in this meta-analysis.

Database	Search Strings with Medical Subject Headings
PubMed	(male OR male OR man OR men OR males OR metabolic syndrome x OR insulin resistance syndrome OR metabolic syndrome OR metabolic syndrome x OR syndrome x, metabolic OR met) AND (bone density OR bone density OR bone mineral density OR density, bone OR osseous density OR osteoporosis OR decalcification, pathological OR endocrine osteoporosis OR osteoporosis OR osteoporotic decalcification) AND (observational study OR non experimental studies OR non experimental study OR nonexperimental studies OR nonexperimental study OR observation studies OR observation study OR observational studies OR observational studies as topic OR observational study OR observational study as topic)
Cinahl	(male OR male OR man OR men OR males OR metabolic syndrome x OR insulin resistance syndrome OR metabolic syndrome OR metabolic syndrome x OR syndrome x, metabolic OR met) AND (bone density OR bone density OR bone mineral density OR density, bone OR osseous density OR osteoporosis OR decalcification, pathological OR endocrine osteoporosis OR osteoporosis OR osteoporotic decalcification) AND (observational study OR non experimental studies OR non experimental study OR nonexperimental studies OR nonexperimental study OR observation studies OR observation study OR observational studies OR observational studies as topic OR observational study OR observational study as topic)
Embase	(‘male’/exp OR ‘male’ OR ‘man’ OR ‘men’ OR ‘males’ OR ‘metabolic syndrome x’/exp OR ‘insulin resistance syndrome’ OR ‘metabolic syndrome’ OR ‘metabolic syndrome x’ OR ‘syndrome x, metabolic’ OR ‘met’/exp) AND (‘bone density’/exp OR ‘bone density’ OR ‘bone mineral density’ OR ‘density, bone’ OR ‘osseous density’ OR ‘osteoporosis’/exp OR ‘decalcification, pathological’ OR ‘endocrine osteoporosis’ OR ‘osteoporosis’ OR ‘osteoporotic decalcification’) AND (‘observational study’/exp OR ‘non experimental studies’ OR ‘non experimental study’ OR ‘nonexperimental studies’ OR ‘nonexperimental study’ OR ‘observation studies’ OR ‘observation study’ OR ‘observational studies’ OR ‘observational studies as topic’ OR ‘observational study’ OR ‘observational study as topic’)
Web of Science	(male OR man OR men OR males OR metabolic syndrome x OR insulin resistance syndrome OR metabolic syndrome OR metabolic syndrome x OR syndrome x, metabolic OR met) AND (bone density OR bone density OR bone mineral density OR density, bone OR osseous density OR osteoporosis OR decalcification, pathological OR endocrine osteoporosis OR osteoporosis OR osteoporotic decalcification) AND observational study)

**Table 2:** Study characteristics.

No	Study Description		Number of Patients	Age (Years; Mean ± SD)	Weight (kg; Mean ± SD)	BMI (kg/m^2^; Mean ± SD)	TC (mg/dL; Mean ± SD)	TC (mg/dL; Mean ± SD)	HDL-C (mg/dL; Mean ± SD)	LDL-C (mg/dL; Mean ± SD)	FPG (mg/dL) Unit	WC (cm; Mean ± SD)	SBP (mmHg; Mean ± SD)	DBP (mmHg; Mean ± SD)
	Reference	Country												
1	Bagherzadeh et al., 2020 [39]	Iran	442	69.08 ± 6.2	ND	28.19 ± 3.72	174.3 ± 43.98	174.78 ± 78.18	37.37 ± 8.5	102.44 ± 37.6	122.33 ±	103 ± 9.74	144.3 ± 18.3	83.72 ± 48.54
2	Da Rocha et al., 2013 [40]	Brazil	23	58.9 ± 13.96	80.71 ± 13.57	31.23 ± 5.35	ND	ND	ND	ND	ND	ND	ND	ND
3	Kim et al., 2019 [41]	South Korea	1080	64.6 ± 9.1	70.9 ± 9.1	25.5 ± 2.6	189.8 ± 37	176.1 ± 125.3	45.8 ± 11.2	112.4 ± 35.9	107.5 ± 24.4	ND	129.9 ± 16.9	80.4 ± 10.2
4	Laurent et al., 2016 [42]	Belgium	975	60.7 (ME*) ± (52–70.4) (IQR*)	91.7 (ME*) ± 82.9–101.1 (IQR*)	30.4 (ME*) ± 28.1–32.8 (IQR*)	ND	177.0 (ME*) ± (124–239) (IQR*)	46 (ME*) ± (39–54) (IQR*)	ND	106 (ME*) ± (99–121) (IQR*)	106.5 (ME*) ± (102.2–113.0) (IQR*)	150.0 (ME*) ± (139.0–164.0) (IQR*)	90.0 (ME*) ± (82.0–98.0) (IQR*)
5	Loke et al., 2018 [29]	Taiwan	691	60.1 ± 7.5	71.1 ± 10.4	25.1 ± 3.3	191.9 ± 36.8	137.9 ± 87.5	52.1 ± 13.5	ND	106.3 ± 29.4	88.2 ± 9.4	134.5 ± 19.8	88.1 ± 11
6	Wani et al., 2019 [37]	Saudi Arabia	243	58.1 ± 9.4	ND	29.9 ± 5.2	189 ± 50	168 (ME*) ± (124-248) (IQR*)	43 ± 12	ND	166 (ME*) ± (117–254) (IQR*)	103.7 ± 14.2	131.1 ± 13.1	79.5 ± 8.2
7	Yakout et al., 2019 [38]	Saudi Arabia	79	39.1 ± 13.2	ND	32.7 ± 4.4	ND	159 (ME*) ± (124-248) (IQR*)	37 ± 8	ND	105 (ME*) ± (92–121) (IQR*)	108.2 ± 11.9	125.3 ± 14.2	84.1 ± 10.6

ME*—median; IQR*—interquartile range; ND—no data; BMI—body mass index; TC—total cholesterol; HDL-C—high density cholesterol; LDL-C—low density cholesterol; FPG—fasting plasma glucose; WC—waist circumference; SBP—systolic blood pressure; DBP—diastolic blood pressure.

**Table 3:** Effect sizes in this meta-analysis.

Time Point	Number of Studies	Point Estimate	SE	Variance	Lower Limit	Upper Limit	Test Z (z Value)	Test Z (*p* Value)	Q Value	df (Q)	*p* Value	Heterogeneity (I2)
**Femoral neck BMD**												
Fixed	4	0.798424287	2.67 × 10^−03^	7.13 × 10^−06^	0.793192401	0.803656173	299.1049353	0	693.6957044	3	0	99.56753372
Random	4	0.845577473	4.56 × 10^−02^	2.08 × 10^−03^	0.756288363	0.934866583	18.56106968	0				
**Lumbar spine BMD**												
Fixed	4	1.032933208	3.54 × 10^−03^	1.25 × 10^−05^	1.026002919	1.039863496	292.1252127	0	710.4211291	3	0	99.57771526
Random	4	1.019173789	6.05 × 10^−02^	3.66 × 10^−03^	0.900574634	1.137772945	16.8428174	0				
**Lumbar spine T-score**												
Fixed	3	−0.571685371	4.60 × 10^−02^	2.12 × 10^−03^	−0.661875484	−0.481495258	−12.42356506	0	38.32124064	2	4.77 × 10^−09^	94.78096229
Random	3	−0.918075893	0.252230741	6.36 × 10^−02^	−1.412439061	−0.423712725	−3.63982554	2.73 × 10^−04^				

## Data Availability

The data presented in this study are available on request from the corresponding author.

## Supplementary Materials

The following supporting information can be downloaded at: https://www.mdpi.com/article/10.3390/biomedicines11071915/s1, Supplementary Figure S1. Funnel plots for the tested parameters, Supplementary Figure S2. Scatterplots for the tested parameters.
