# Supplementary figures and images for: The Impact of Metabolic Syndrome on Bone Mass in Men: Systematic Review and Meta-Analysis
Source: Biomedicines. 2023 Jul 6;11(7):1915. doi: 10.3390/biomedicines11071915 (PMC10377465; doi:10.3390/biomedicines11071915)

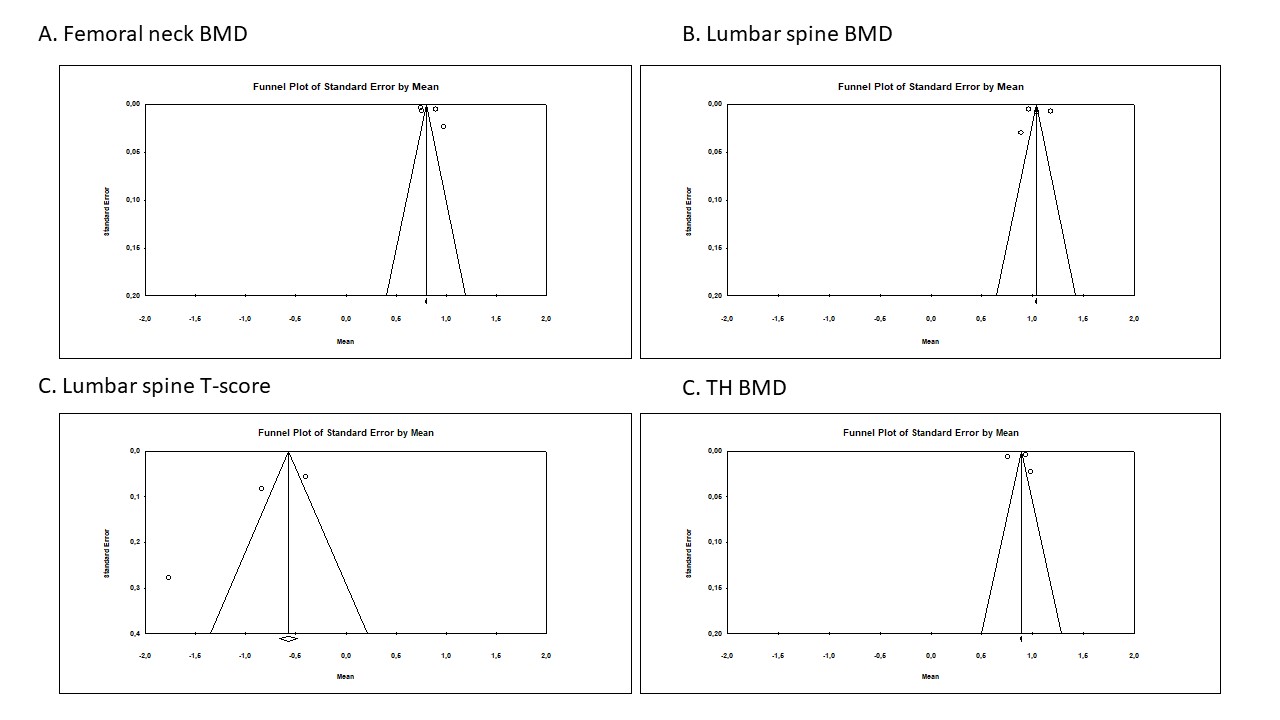

Supplement: Supplementary file 1 [file biomedicines-11-01915-s001.zip › Supplementary Figure S1.jpg]

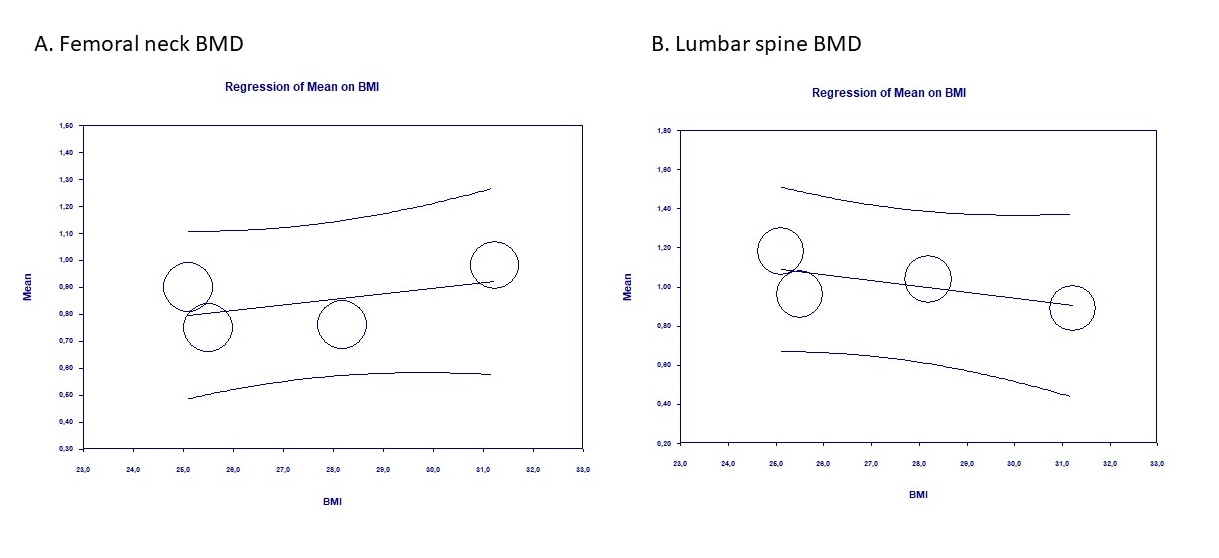

Supplement: Supplementary file 1 [file biomedicines-11-01915-s001.zip › Supplementary Figure S2.jpg]
